# Association of Obesity and Luminal Subtypes in Prognosis and Adjuvant Endocrine Treatment Effectiveness Prediction in Chinese Breast Cancer Patients

**DOI:** 10.3389/fonc.2022.862224

**Published:** 2022-05-05

**Authors:** Yiwei Tong, Siyi Zhu, Weiguo Chen, Xiaosong Chen, Kunwei Shen

**Affiliations:** Department of General Surgery, Comprehensive Breast Health Center, Ruijin Hospital, Shanghai Jiao Tong University School of Medicine, Shanghai, China

**Keywords:** breast cancer, obese, luminal subtype, prognosis, aromatase inhibitor

## Abstract

**Purpose:**

To evaluate the influence of obesity on clinicopathological characteristics of breast cancer; to explore the effect of obesity on the prognosis and performance of endocrine therapy in breast cancer patients.

**Methods:**

Patients with luminal/HER2-negative early breast cancer were included and categorized into the non-obese (BMI<28kg/m^2^) and obese (BMI≥28kg/m^2^) groups according to body mass index (BMI). Clinicopathological characteristics and treatment modalities were compared between groups. Interaction of adjuvant endocrine therapy with obesity was analyzed.

**Results:**

A total of 2,875 patients were included: 2,598 non-obese and 277 obese. A higher rate of patients with comorbidities (OR: 2.83, 95%CI 2.13-3.74, *P*<0.001) or PR-positive tumor (OR: 1.63, 95%CI 1.03-2.58, *P*=0.037) were identified in the obese group. Obesity was not associated with disease recurrence (*P*=0.839) or overall survival (*P*=0.140) in the whole population. Subgroup analysis did show an association with worse relapse-free survival (RFS, HR 3.48, 95%CI 1.31-9.22, *P*=0.012) and overall survival (OS, HR 4.67, 95%CI 1.28-16.95, *P*=0.019) in luminal A breast cancer. These results could not be reproduced in the luminal B subtype with a RFS (HR 0.78, 95%CI 0.41-1.49, *P*=0.454) or OS (HR 1.17, 95%CI 0.50-2.74, *P*=0.727). Furthermore, obesity did not impact endocrine therapy effectiveness in Tamoxifen or the aromatase inhibitor group (RFS: interact *P*=0.381; OS: interact *P*=0.888).

**Conclusions:**

The impact of obesity on prognosis interacted with luminal subtype status in Chinese breast cancer patients which was not related with endocrine treatment modality.

## Introduction

Breast cancer is the most commonly diagnosed cancer among women (1), with luminal tumors accounting for 70% of all cases (2). Obesity has been widely recognized as a risk factor for postmenopausal breast cancer but rather protective for premenopausal women from developing breast cancer (3–5). Meanwhile, obesity is associated with impaired survival, regardless of menopausal status (3, 4). Substantial evidence has suggested that obese women with early stage disease have an increased risk of recurrence (6–9) and reduced survival (6–12) compared to normal-weight women with early-stage disease. Studies have also identified obesity as a risk factor for contralateral breast cancer, as well as second primary cancers (13, 14). Furthermore, the association between obesity and outcomes of breast cancer varied by tumor estrogen receptor (ER) status (15). The prospective POSH study, which enrolled 2,956 patients aged ≤40 at breast cancer diagnosis, revealed that obesity is an independent prognostic factor for ER-positive but not ER-negative breast cancer (15). Regarding molecular subtypes, the association between obesity and prognosis was still in controversy (15–17). Increasing body mass index (BMI) was found to be associated with inferior outcomes in luminal/human epidermal growth factor receptor 2 (HER2)-negative disease but not other subtypes (7, 16, 18).

Obesity has been associated with increased local and circulating estrogen production due to high expression of the aromatase in the breast and peripheral fat (19, 20). Aromatase inhibitors (AIs) can inhibit the conversion of androgens to estrogens in adipose tissue, indicating AIs may be less effective in obese postmenopausal patients (21). Retrospective analysis from ATAC and ABCSG-12 trials found that obese patients treated with anastrozole had worse prognosis than those without obesity. This was not the case in the tamoxifen treatment group (22, 23).

Therefore, the objectives of our study are to explore whether obesity is prognostic and predictive in Chinese breast cancer survivors. We also searched for potential clinicopathological features, for instance, a luminal subtype that might influence the impact of obesity on survival and performance of endocrine therapy in breast cancer patients.

## Methods

### Patients

Patients included in the study underwent radical surgery in the Comprehensive Breast Health Center, Ruijin Hospital, Shanghai Jiao Tong University between January 2009 and June 2018. The inclusion criteria were female patients diagnosed with invasive luminal/HER2-negative invasive breast cancer. Luminal-like was defined as ER and/or progesterone receptor (PR) positive. The exclusion criteria included: missing values for BMI, missing information for adjuvant therapy, receiving neoadjuvant treatment, having previous malignancy in medical history, and having stage IV of disease. This study was performed in accordance with the terms of the Declaration of Helsinki and approved by the Ethical Committee Review Board of Institution.

### Clinicopathological Characteristics and Follow Up

Clinicopathological information was retrieved from Shanghai Jiao Tong University Breast Cancer Database (SJTU-BCDB), including BMI, age, menopausal status, comorbidities evaluated by Charlson Comorbidity Index (CCI), type of surgery, histopathological type, histological grade, expression of ER, PR, Ki-67, HER2 status, tumor size, lymph node involvement, and adjuvant treatment. Included patients were less than 60 years of age, less than 60 years of age and amenorrheic for at least 36 months, or with prior bilateral oophorectomy who were considered postmenopausal.

BMI at baseline, collected at the first day after hospitalization for surgery, was calculated as weight in kilograms divided by the square of the height in meters and was used in the classification of obesity (BMI ≥ 28 kg/m^2^) and non-obesity (BMI < 28 kg/m^2^) (24). Hormone receptor (HR) positivity was defined if there was at least 1% staining in tumor nuclei. ER expression cut-off was set at 50% since the St. Gallen International Expert Consensus on the Primary Therapy of Early Breast Cancer considered tumor cell HR staining≥50% as highly endocrine-responsive tumors (25). Status of HER2 was determined according to the 2018 ASCO/CAP (American Society of Clinical Oncology/College of American Pathologists) guideline for HER2 testing (26). Invasive luminal/HER2-negative breast cancers were classified into two subtypes: luminal A and luminal B/HER2-negative according to the 2015 St. Gallen Consensus (27). Luminal A is defined as HR-positive, HER2-negative, PR ≥ 20%, and Ki-67 < 14%. Luminal B/HER2-negative is defined as HR-positive, HER2-negative, and at least one of the following: Ki-67 ≥ 14% or PR negative or < 20%.

Adjuvant treatment was decided for each patient through multidisciplinary discussion. Patients would receive treatment reminders from a center-specific smart-phone based application (28) and from breast cancer-specific nurses. Patient’s adherence to treatment was evaluated through regular prescription records. Patients were considered non-adherent if they received treatment inconsistent with multidisciplinary team recommendation or if they spontaneously stopped treatment regardless of physician recommendation. Follow-up was accomplished annually by breast cancer specific nurses and assistants in our center through medical records reviews and/or regular phone calls. Follow-up information regarding recurrence and survival status were updated to June 2020. Relapse-free survival (RFS) and overall survival (OS) were primary outcomes. RFS was defined as the interval between surgery and ipsilateral ductal carcinoma *in situ* (DCIS), ipsilateral invasive breast tumor recurrence, locoregional invasive recurrence, distant recurrence, and death from any cause. OS was defined as the interval between surgery and death from any cause (29).

### Statistical Analysis

Categorical variables were compared using Chi-square tests or Fisher’s exact tests to find out the difference of clinicopathological characteristics and receipt of adjuvant treatment between obese and non-obese patients. Multivariate logistic regression was applied to analyze the association between obesity and clinicopathological characteristics. Kaplan–Meier curve and log-rank test were performed to compare disease outcomes between obese and non-obese patients stratified by luminal subtypes. Subgroup analysis and forest plots were conducted using stratified Mantel-Haenszel test to estimate hazard ratio (HR) with 95%CI (confidence interval). Cox proportional hazards regression model was applied to determine the association between obesity and survival outcomes in different subgroups. Two-tailed *P* value <0.05 was considered statistically significant. All statistical analyses were carried out using SPSS version 26 (SPSS, Inc., Chicago, IL, USA).

## Results

### Clinicopathological Characteristics and Adjuvant Treatment

**Figure 1:**
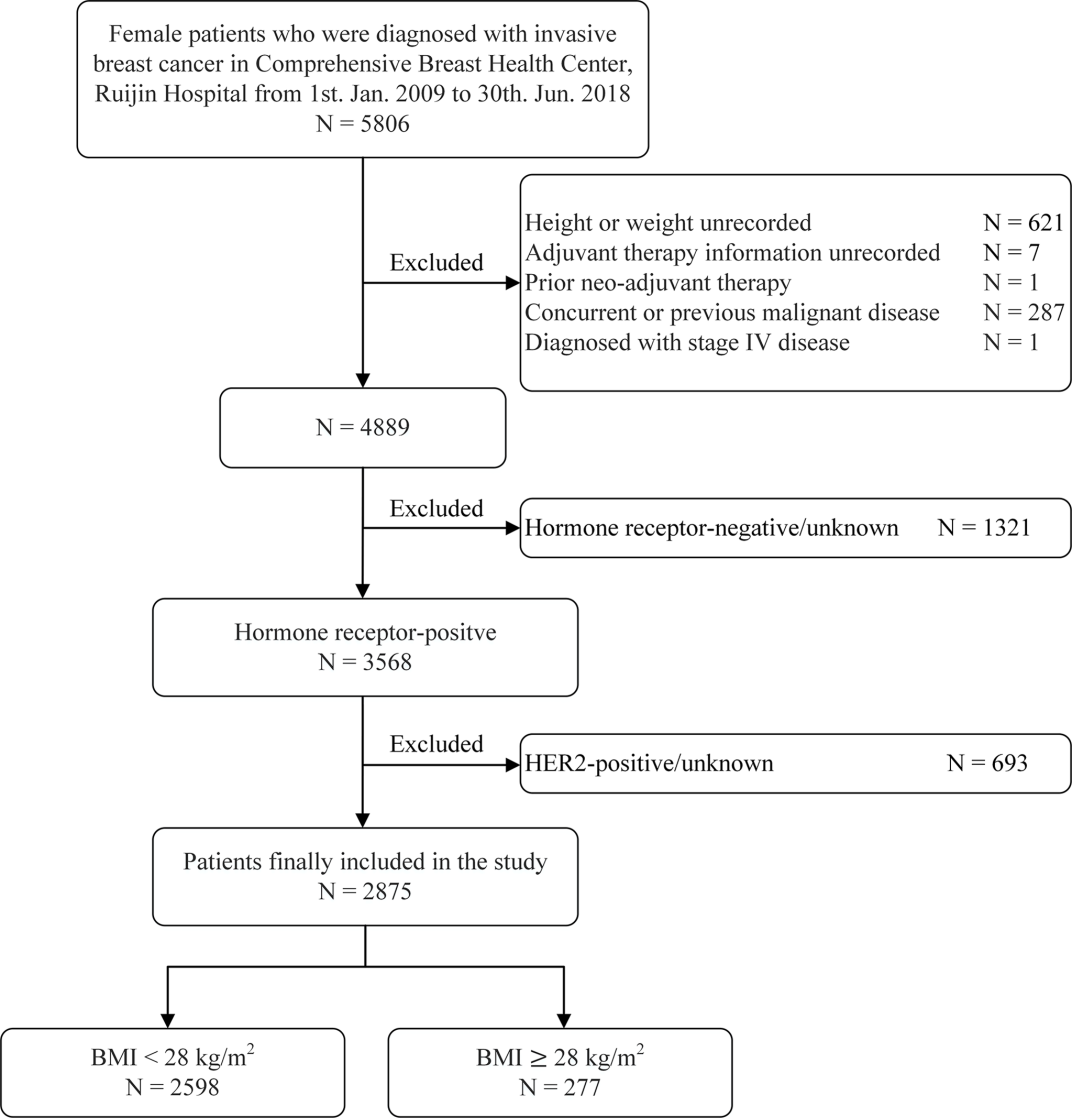
Study flow chart. BMI, body mass index; HER2, human epidermal growth factor receptor 2.

A total of 2,875 luminal breast cancer patients were included (**Figure 1**). Clinicopathological characteristics and adjuvant treatments according to BMI groups were listed in **Tables 1**,** 2**. The mean age of the study population was 56.41 ± 12.71 years old and 66.6% of patients were aged ≥50 years old. There were 919 (32.0%) patients with at least one comorbidity by CCI, for instance diabetes, chronic kidney diseases, peripheral vascular disease, etc. Only 7.6% of patients had lymphovascular invasion. A total of 1,851 (64.4%) patients had tumors ≤ 2.0 cm. Exactly 1,882 (65.5%) patients presented with node negative disease. There were 1,743 (60.6%) and 665 (23.1%) with grade I-II and III tumors, respectively, leaving the remaining 467 (16.2%) patients histologic grade unknown. Invasive ductal carcinoma (IDC) was diagnosed in 2,407 (83.7%) patients. The proportions of patients with ER ≥ 50%, PR-positive, and Ki−67 ≥ 14% were 91.5%, 85.2%, and 54.4% respectively. Moreover, 926 (32.2%), and 1,949 (67.8%) patients had luminal A, and luminal B/HER2-negative tumors. The clinicopathological features by luminal subtype was presented in **Supplementary Table S1**.

**Table 1:** Distribution of clinicopathological characteristics in the whole population and different BMI groups.

Characteristics	Whole population (N = 2875) N (%)	BMI < 28 kg/m^2^ (N= 2598) N (%)	BMI ≥ 28 kg/m^2^ (N = 277) N (%)	*P* value
Age at diagnosis (year), mean ± SD	56.41 ± 12.71	55.85 ± 12.72	61.57 ± 11.46	< 0.001
Age at diagnosis (year)				
< 50	961 (33.4)	917 (35.3)	44 (15.9)	< 0.001
≥ 50	1914 (66.6)	1681 (64.7)	233 (84.1)	
Menopausal status				< 0.001
Pre/peri-menopausal	1109 (38.6)	1051 (40.5)	58 (20.9)	
Post-menopausal	1766 (61.4)	1547 (59.5)	219 (79.1)	
Comorbidities				< 0.001
None	1956 (68.0)	1844 (71.0)	112 (40.4)	
≥ 1	919 (32.0)	754 (29.0)	165 (59.6)	
Lymphovascular invasion				0.811
Negative	2657 (92.4)	2400 (92.4)	257 (92.8)	
Positive	218 (7.6)	198 (7.6)	20 (7.2)	
Pathologic tumor size				0.172
≤ 2cm	1851 (64.4)	1683 (64.8)	168 (60.6)	
> 2cm	1024 (35.6)	915 (35.2)	109 (39.4)	
Pathologic nodal status				0.756
Negative	1882 (65.5)	1696 (65.3)	186 (67.1)	
Positive	967 (33.6)	879 (33.8)	88 (31.8)	
Unknown	26 (0.9)	23 (0.9)	3 (1.1)	
Histological grade				0.465
I-II	1743 (60.6)	1568 (60.4)	175 (63.2)	
III	665 (23.1)	601 (23.1)	64 (23.1)	
Unknown	467 (16.2)	429 (16.5)	38(13.7)	
Histopathological type				0.297
Non-IDC	468 (16.3)	429 (16.5)	39 (14.1)	
IDC	2407 (83.7)	2169 (83.5)	238 (85.9)	
ER expression				0.005
< 50%	244 (8.5)	233 (9.0)	11 (4.0)	
≥ 50%	2631 (91.5)	2365 (91.0)	266 (96.0)	
PR status				0.005
Negative	425 (14.8)	400 (15.4)	25 (9.0)	
Positive	2450 (85.2)	2198 (84.6)	252 (91.0)	
Ki-67 expression				0.323
< 14%	1310 (45.6)	1176 (45.3)	134 (48.4)	
≥ 14%	1565 (54.4)	1422 (54.7)	143 (51.6)	
Molecular subtype				0.046
Luminal A	926 (32.2)	822 (31.6)	104 (37.5)	
Luminal B/HER2-negative	1949 (67.8)	1776 (68.4)	173 (62.5)	

BMI, body mass index; SD, standard deviation; IDC, invasive ductal carcinoma; ER, estrogen receptor; PR, progesterone receptor; HER2, human epidermal growth factor receptor 2.

**Table 2:** Distribution of treatment in the whole population and different BMI groups.

Characteristics	Whole population (N = 2875) N (%)	BMI < 28 kg/m^2^ (N= 2598) N (%)	BMI ≥ 28 kg/m^2^ (N = 277) N (%)	*P* value
Surgery of the breast				0.778
Breast-conserving surgery	1029 (35.8)	932 (35.9)	97 (35.0)	
Mastectomy	1846 (64.2)	1666 (64.1)	180 (65.0)	
Surgery of the axilla				0.499
SLNB	1709 (59.4)	1547 (59.5)	162 (58.5)	
ALND	1140 (39.7)	1029 (39.6)	111 (40.1)	
Unknown	26 (0.9)	22 (0.8)	4 (1.4)	
Chemotherapy				0.027
No	1262 (43.9)	1123 (43.2)	139 (50.2)	
Yes	1613 (56.1)	1475 (56.8)	138 (49.8)	
Radiotherapy				0.483
No	1354 (47.1)	1218 (46.9)	136 (49.1)	
Yes	1521 (52.9)	1380 (53.1)	141 (50.9)	
Endocrine therapy				0.154
No	137 (4.8)	119 (4.6)	18 (6.5)	
Yes	2738 (95.2)	2479 (95.3)	259 (93.5)	
Ovarian function suppression				< 0.001
No	2701 (93.9)	2427 (93.4)	274 (98.9)	
Yes	174 (6.1)	171 (6.6)	3 (1.1)	

BMI, body mass index; SLNB, sentinel lymph node biopsy; ALND, axillary lymph node dissection.

In terms of surgical approach, 1,029 (35.8%) patients received breast-conserving surgery (BCS) and 1,709 (59.4%) patients received sentinel lymph node biopsy (SLNB). Regarding adjuvant treatment, the proportions of patients who underwent adjuvant chemotherapy, radiotherapy, endocrine therapy, and ovarian function suppression (OFS) treatment were 56.1%, 52.9%, 95.2%, and 6.1%, respectively. A total of 2,738 patients received adjuvant endocrine therapy. According to the first endocrine therapy agent received, 1,872 patients received AI treatment (including 118 patients who received OFS simultaneously) and 866 patients received selective estrogen receptor modulators (SERM) treatment (including 56 patients who received OFS simultaneously). A total of 174 patients received OFS.

### Association of Clinicopathological Characteristics and Obesity

The median BMI of the whole population was 23.15 (21.30-25.39) kg/m^2^: 2598 (90.37%) with BMI < 28 kg/m^2^ and the remaining 277 (9.63%) with BMI ≥ 28 kg/m^2^. Univariate analysis found that age, menopausal status, comorbidities, ER expression, PR positivity, and luminal subtypes were significantly different between two BMI groups (all *P* < 0.05, **Table 1**). Lymphovascular invasion, tumor size, node involvement, grade 3 tumors, histologic type, and Ki-67 percentage were not different between the non-obese and obese patients (*P* > 0.05).

Multivariate analysis demonstrated that only comorbidities and PR positivity were independently associated with obesity (**Table 3**). Obese patients were more likely to be diagnosed with concomitant comorbidities [odds ratio (OR) 2.83, 95%CI 2.13-3.74, *P* < 0.001] and slightly more PR-positive tumors (OR 1.63, 95%CI 1.03-2.58, *P* = 0.037). It also revealed the trend for obese patients to be older than non-obese patients (OR 1.71, 95%CI 0.97-3.02, *P* = 0.066).

**Table 3:** Multivariate analysis of factors associated with obesity.

Characteristics	OR (95%CI)	*P* value
Age at diagnosis (year)		0.066
≥ 50 vs < 50	1.71 (0.97-3.02)	
Menopausal status		0.741
Post- vs pre/peri-menopausal	1.09 (0.65-1.84)	
Comorbidities		< 0.001
≥ 1 vs 0	2.83 (2.13-3.74)	
ER expression		0.221
≥ 50% vs < 50%	1.50 (0.78-2.88)	
PR status		0.037
Positive vs negative	1.63 (1.03-2.58)	
Molecular subtype		0.552
luminal B vs luminal A	0.92 (0.70-1.21)	

OR, odds ratio; CI, confidence interval; ER, estrogen receptor; PR, progesterone receptor.

### Obesity and Clinical Outcomes

**Figure 2:**
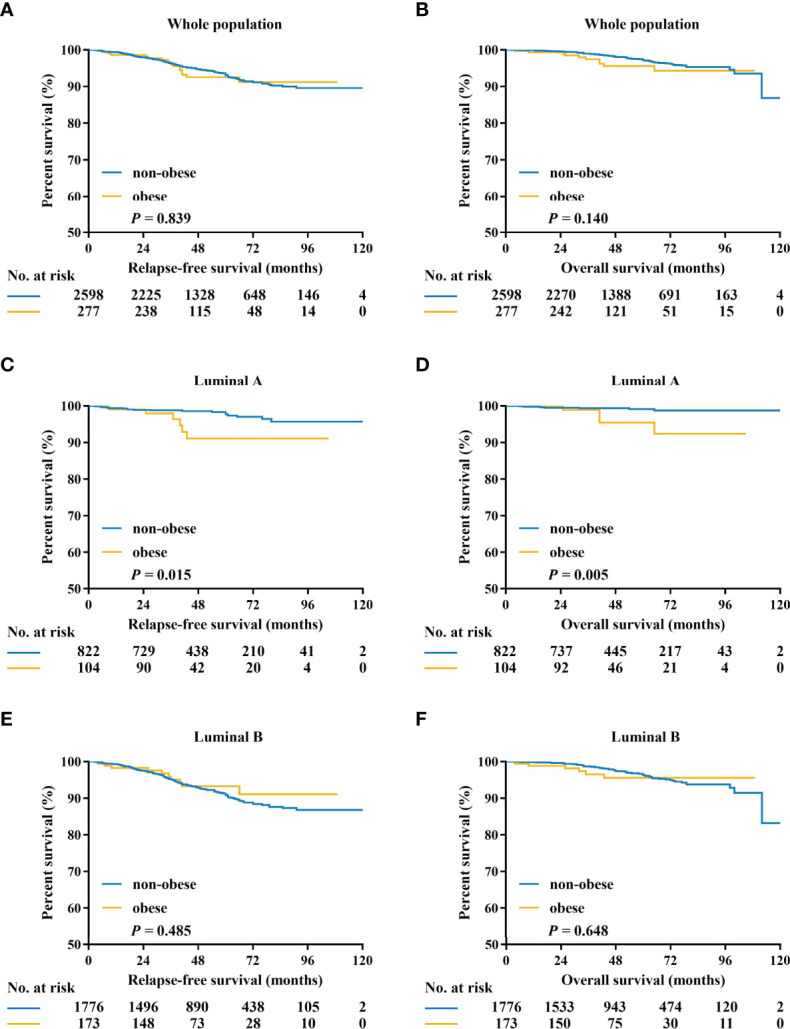
Kaplan–Meier estimates of relapse-free survival and overall survival in patients with HR-positive/HER2-negative tumors **(A, B)**, luminal A tumors **(C, D)**, and luminal B tumors **(E, F)**. HR, hormone receptor; HER2, human epidermal growth factor receptor 2.

After a median follow-up of 50 (range 4-127) months, 170 RFS events, and 75 deaths have been recorded. The 5-year RFS and OS were 88.2% and 93.6% in the whole population. Pathological tumor size, node status, histological grade, PR expression, and adjuvant endocrine therapy were independent predictors for RFS in luminal patients, while luminal subtype was not independently associated with survival (*P*=0.075, **Supplementary Table S2**). There was no significant difference between non-obese and obese patients in terms of RFS (5-year RFS 88.0% vs 90.3%, *P* = 0.839) and OS (5-year OS 93.6% vs 93.4%, *P* = 0.140; **Figures 2A, B**; **Supplementary Table S2**).

**Figure 3:**
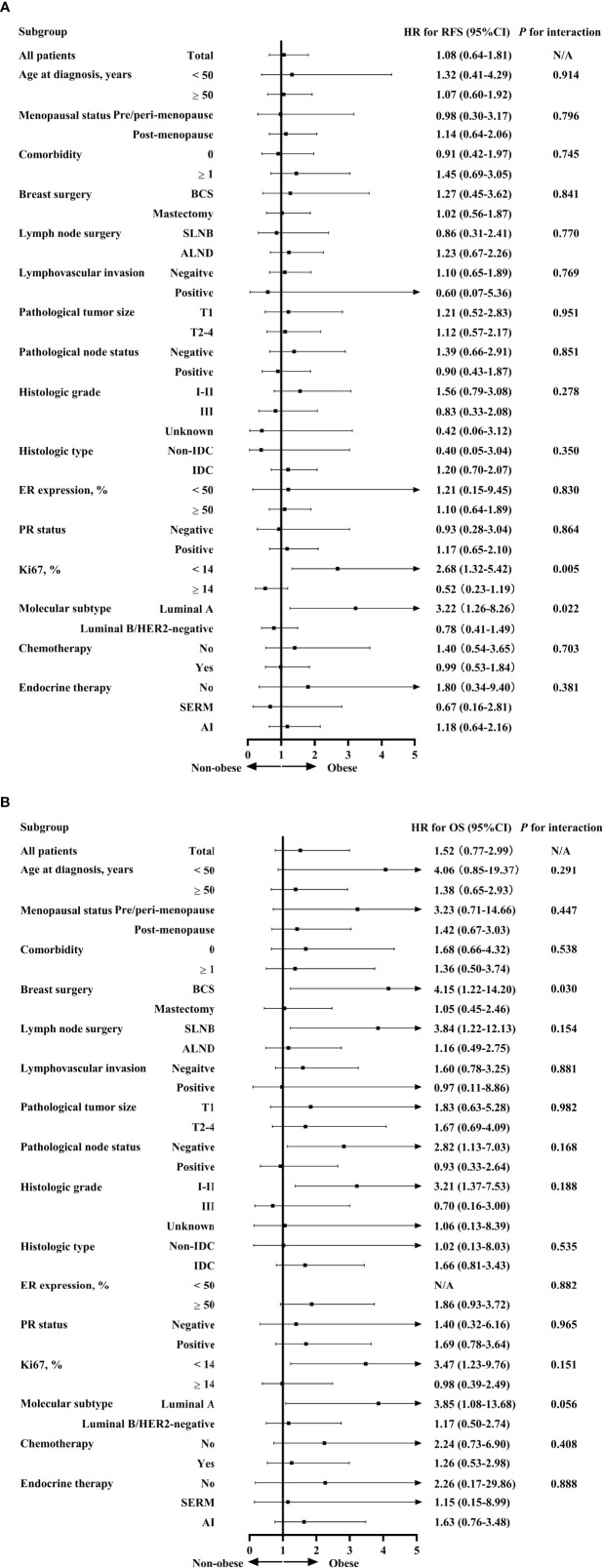
Interaction between BMI and clinicopathological characteristics in predicting RFS **(A)** and OS **(B)**. BMI, body mass index; RFS, relapse-free survival; OS, overall survival; HR, hazard ratio; CI, confidence interval; BCS, breast conservative surgery; SLNB, sentinel lymph node biopsy; ALND, axillary lymph node dissection; IDC, invasive ductal carcinoma; ER, estrogen receptor; PR, progesterone receptor; HER2, human epidermal growth factor receptor 2; SERM, selective estrogen receptor modulators; AI, aromatase inhibitors.

Exploratory subgroup analysis was then done to evaluate prognosis difference according to different clinicopathological subtypes between the obese and non-obese BMI groups (**Figure 3**). As shown in the forest plot, obesity had a different impact on prognosis for the two luminal subtypes (interact *P* = 0.022 for RFS, interact *P* = 0.056 for OS; **Figures 3A, B**). For patients with luminal A disease, those obese reported a significant impaired RFS (5-year RFS: 91.1% vs 94.6%, *P* = 0.015; **Figure 2C**) and OS (5-year OS: 90.2% vs 98.5%, *P* = 0.005; **Figure 2D**) compared to non-obese patients. Multivariate analysis demonstrated that obesity was an independent risk factor for luminal A patients, with an adjusted HR of 3.48 (95%CI 1.31-9.22) for RFS and 4.67 (95%CI 1.28-16.95) for OS. Meanwhile, obesity had no significant impact on RFS (univariate *P* = 0.485; **Figure 2E**; multivariate HR 0.78, 95%CI 0.41-1.49, *P* = 0.454; **Supplementary Table S4**) or OS (univariate *P* = 0.648; **Figure 2F**; multivariate HR 1.17, 95%CI 0.50-2.74, *P* = 0.727; **Supplementary Table S5**) in luminal B population.

Apart from luminal subtype, no other clinicopathological feature had interaction with obesity in prognosis prediction (**Figure 3**). For instance, when stratified by menopausal status, obesity was not associated with survival in either pre/peri-menopausal patients or post-menopausal patients in terms of RFS (interact *P* = 0.796) or OS (interact *P* = 0.447).

### Association of Obesity and Prognosis According to Endocrine Treatment

**Figure 4:**
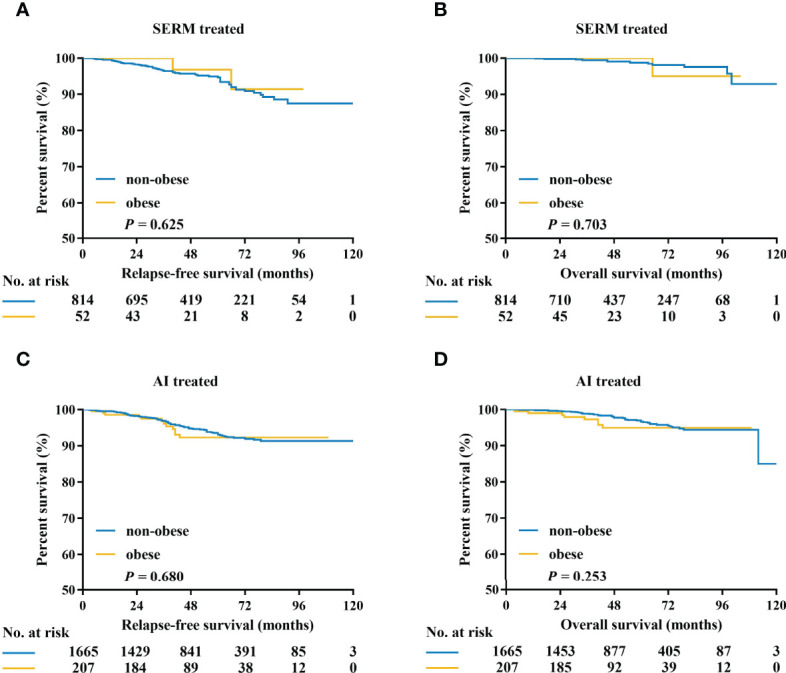
Kaplan–Meier estimates of relapse-free survival and overall survival in patients treated with SERM **(A, B)**, and AI **(C, D)**. SERM, selective estrogen receptor modulators; AI, aromatase inhibitors.

The adherence to endocrine therapy was 91.8% (1,719/1,872) for AI and 92.5% (801/866) for SERM (*P*=0.549). Kaplan–Meier curves of RFS and OS according to obesity status in patients treated with SERM or AI are listed in **Figure 4**. There was no interaction between obesity and endocrine treatment modality in predicting RFS (interact *P* = 0.381) and OS (interact *P* = 0.888). In the subgroup of patients on SERM treatment (with or without OFS), the obese patients showed no significant differences in 5-year RFS (87.0% vs 85.3%, *P* = 0.625) or 5-year OS (90.9% vs 95.9%, *P* = 0.703) compared with the non-obese (**Figures 4A, B**). Regarding patients receiving AI treatment (with or without OFS), there was no difference in OS or RFS between obese and non-obese patients (5-year RFS: 92.2% vs 90.1%, *P* = 0.680, **Figure 4C**; 5-year OS: 94.9% vs 92.9%, *P* = 0.253; **Figure 4D**). Patients receiving OFS showed similar RFS and OS between the two BMI groups (data not shown).

## Discussion

Our current study included 2,875 Chinese luminal breast cancer patients. We have found that obesity was related to the patient’s comorbidity and PR status. We also concluded that obese patients with luminal A breast cancer have significantly shorter OS and RFS. This relation was not significant in luminal B breast cancer. Our study is one of the few to investigate the impact of obesity on prognosis of breast cancer patients according to luminal subtypes in the Chinese population, which may provide an insight into how obesity adversely influences clinical outcomes.

Nearly one-tenth of our study population were obese, which was relatively lower than the Western population (30). In our study, we found that obese patients tended to be elder and more likely to have a medical history of comorbidities compared to non-obese patients, which is consistent with previous studies (6–8, 17, 31). Interestingly, our cohort found that obese patients had a slightly higher proportion of PR-positive tumors compared to non-obese patients, which was similar to the findings of Ladoire et al. (31). A preclinical study with obese rats with breast cancer induced by high-fat diet and 1-methyl-1-nitrosourea demonstrated that obesity enhanced the tumor expression of PR. Obesity-induced PR expression in tumors was thought to be an adaption to extreme metabolic changes and loss of sex hormone production during menopause (32). Additionally, previous studies reported that obesity was related with more advanced and biologically adverse tumors, such as larger tumor size, more lymph node involvement, higher proportion of ER-negative, triple negative tumors, grade III, or high Ki-67 index, and this could not be reproduced in our study (16, 33, 34), which may be explained by our study only including the luminal population. Molecular subtypes were similarly distributed in obese and non-obese patients in our study. Whereas in the PAM50 testing dated from the CALGB 9741 study, obese breast cancer patients had a significantly larger share of luminal B breast cancers (35), possibly explained by ethnic background difference or different molecular subtype detecting methods. In terms of adjuvant systemic therapy, obese patients were less likely to receive chemotherapy, which may be contributed to the elder age and the relatively worse baseline performance status (36, 37). Moreover, obese patients would also be more likely to receive insufficient dosage of treatment according to actual body surface area, which would also impact the patient’s disease outcome (38).

With a growing body of evidence exploring the correlation between obesity and clinical outcomes, controversy still exists on the relation between obesity and prognosis in specific molecular subtypes of breast cancer (7, 17, 19, 39). In our current study, we did not find the prognostic value of obesity in the overall luminal/HER2-negative population or in the subgroup of luminal B tumors. Meanwhile, we demonstrated a significant inverse relationship between BMI and disease outcomes in patients with luminal A breast cancer, which was in accordance with previously published literature based on PAM50 subtypes (40, 41). In an analysis based on the LACE and Pathways studies, obesity was adversely correlated with outcomes only among those with luminal A cancers, basically driven by class II/III obesity (BMI ≥ 35 kg/m^2^) (40). Similarly, Kwan et al. also found that the association between BMI and disease outcome was the strongest for luminal A tumors (41). Though obesity has been shown to be related with less favorable characteristics (9, 42, 43), the statistical significance of survival outcome still existed in luminal A patients after adjusting for other clinicopathological factors. Possible explanations for such difference between luminal A and luminal B tumors might be as follows. Firstly, increased endogenous estrogen with obesity would fuel the growth of ER-positive tumors but the effect would be less obvious for luminal B tumors which were also hormone receptor-positive but to a lesser extent (40). In addition, luminal B disease had a more aggressive nature compared to luminal A disease with increased proliferation and less hormone receptor expression, which would overlap and mask the effect of obesity. In addition, we showed in the univariate model that nodal status was associated with RFS in Luminal A patients (**Supplementary Table S3**). However, node status was no longer significantly related to prognosis in Luminal A patients in the multivariate model (**Table 4**), possibly due to the effect of chemotherapy, which suggests that standard adjuvant chemotherapy brings substantial benefit for node positive Luminal patients.

**Table 4:** Multivariate analysis of factors associated with RFS and OS in luminal A patients.

	RFS		OS	
Variables	HR (95%CI)	*P* value	HR (95%CI)	*P* value
Age : ≥ 50 vs < 50	0.75 (0.31-1.84)	0.528	1.68 (0.35-8.18)	0.520
BMI: obese vs non-obese	3.48 (1.31-9.22)	0.012	4.67 (1.28-16.95)	0.019
Pathologic tumor size: > 2cm vs ≤ 2cm	2.18 (0.94-5.06)	0.071	1.21 (0.33-4.43)	0.774
Pathologic nodal status:		0.176		0.155
positive vs negative	2.08 (0.71-6.08)	0.182	2.04 (0.54-7.73)	0.292
unknown vs negative	4.73 (0.61-36.88)	0.138	7.33 (0.86-62.21)	0.068
Chemotherapy: yes vs no	1.57 (0.53-4.62)	0.412		

RFS, relapse-free survival; OS, overall survival; HR, hazard ratio; CI, confidence interval; BMI, body mass index.

It is widely recognized that obesity has great influence on the endocrine system both in pre- and post-menopausal breast cancer patients (20, 21). Retrospective analysis from the ATAC trial found that tamoxifen was equally effective across all BMI levels, while anastrozole was significantly less effective in obese patients compared to normal weight patients (23). Similarly, results from the ABCSG-12 trial, exclusively including premenopausal patients treated with OFS, showed a worse disease outcome for obese patients treated with AI but not for tamoxifen cohorts (24). Speculation has arisen whether the constant dosage of anastrozole is sufficient for the obese to suppress the estrogen to a relatively low extent. Anastrozole was found to be inferior to tamoxifen for overweight or obese premenopausal patients in the ABCSG-12 trial and equal to tamoxifen for obese postmenopausal patients in the ATAC trial (23, 24). Other large phase III trials did not find any influence of obesity on letrozole (34) or exemestane (44) performance, possibly due to the different potencies of estrogen level suppression ability (45). In our study, we did not observe an effect of obesity on AI or tamoxifen treatment. Studies of large sample size are needed and essential to compare the efficacy of different types of AI for obese patients.

There are certainly several limitations in this study. Firstly, it is a retrospective study from a single institution with possible selection bias. Secondly, we did not take the effect of weight change after diagnosis into account for the lack of relevant information. Weight gain following breast cancer diagnosis has been reported to be associated with worse survival (46, 47). In addition, we did not include the lifestyle covariates like physical activity and diet preference into survival analysis, which have been related with breast cancer outcomes (48–50). Finally, it is important to carry out a comprehensive analysis of additional metabolic parameters other than obesity, for instance, metabolic syndrome, insulin, c-peptide, blood glucose, etc. on patients’ outcomes (51–53), so as to provide a more precise view regarding patient metabolism and cancer survival.

In conclusion, the adverse impact of obesity on luminal-like patients’ survival announced in previous studies could not be reproduced with our cohort of the Chinese luminal-like population. Our study found that obesity was associated with slightly more PR positive tumor and worse clinical outcomes in luminal A subtype but not for luminal B tumor. In patients treated with adjuvant endocrine therapy, obesity had no significant impact on patients’ outcome, regardless of endocrine treatment regimen received, which deserves further clinical evaluation.

## Data Availability Statement

The raw data supporting the conclusions of this article will be made available by the authors, without undue reservation.

## Ethics Statement

The studies involving human participants were reviewed and approved by independent Ethical Committees of Ruijin Hospital, Shanghai Jiao Tong University School of Medicine. The patients/participants provided their written informed consent to participate in this study.

## Author Contributions

Data collection and analysis were performed by SZ. YT, SZ, and WC wrote the first draft of the manuscript. XC made substantial contributions to the conception of the work and revised the manuscript. KS substantively revised the manuscript. All authors have contributed to read and approved the final manuscript for submission.

## Funding

The authors appreciate the financial supports by the National Natural Science Foundation of China (Grant Number: 81772797, 82072897), Shanghai Municipal Education Commission—Gaofeng Clinical Medicine Grant Support (20172007); and Shanghai Sailing Program (21YF1427400). All these financial sponsors had no role in the study design, collection, analysis or interpretation of data.

## Conflict of Interest

The authors declare that the research was conducted in the absence of any commercial or financial relationships that could be construed as a potential conflict of interest.

## Publisher’s Note

All claims expressed in this article are solely those of the authors and do not necessarily represent those of their affiliated organizations, or those of the publisher, the editors and the reviewers. Any product that may be evaluated in this article, or claim that may be made by its manufacturer, is not guaranteed or endorsed by the publisher.

## Abbreviations

AI, Aromatase inhibitor; ASCO, American Society of Clinical Oncology; BCS, Breast-conserving surgery; BMI, Body mass index; CAP, College of American Pathologists; CI, Confidence interval; DCIS, Ductal carcinoma in situ; ER, Estrogen receptor; HER2, Human epidermal growth factor receptor 2; HR, Hormone receptor; HR, Hazard ratio; IDC, Invasive ductal carcinoma; OFS, Ovarian function suppression; OR, Odds ratio; OS, Overall survival; PR, Progesterone receptor; RFS, Recurrence-free survival; SERM, Selective estrogen receptor modulator; SLNB, Sentinel lymph node biopsy.
