# Supplementary material for: Association of Obesity and Luminal Subtypes in Prognosis and Adjuvant Endocrine Treatment Effectiveness Prediction in Chinese Breast Cancer Patients
Source: Front Oncol. 2022 May 5;12:862224. doi: 10.3389/fonc.2022.862224 (PMC9117630; doi:10.3389/fonc.2022.862224)
Supplement: Supplementary file 1 [file Table_1.docx]

**Supplementary Table S1.** Distribution of clinicopathological characteristics in the whole population and different luminal subtypes

| Characteristics | Whole population  (N = 2875)  N (%) | Luminal A  (N= 926)  N (%) | Luminal B  (N = 1949)  N (%) | *P* value |
| --- | --- | --- | --- | --- |
| Age at diagnosis (year), mean ± SD | 56.41 ± 12.71 | 57.72 ± 12.86 | 55.78 ± 12.59 | < 0.001 |
| Age at diagnosis (year) |  |  |  |  |
| < 50 | 961 (33.4) | 310 (33.5) | 651 (33.4) | 0.968 |
| ≥ 50 | 1914 (66.6) | 616 (66.5) | 1298 (66.6) |  |
| BMI, mean ± SD | 23.50 ± 3.27 | 23.62 ± 3.27 | 23.44 ± 3.27 | 0.679 |
| BMI |  |  |  | 0.046 |
| < 28 kg/m^2^ | 2598 (90.4) | 822 (88.8) | 1776 (91.1) |  |
| ≥ 28 kg/m^2^ | 277 (9.6) | 104 (11.2) | 173 (8.9) |  |
| Menopausal status |  |  |  | 0.522 |
| Pre/peri-menopausal | 1109 (38.6) | 365 (39.4) | 744 (38.2) |  |
| Post-menopausal | 1766 (61.4) | 561 (60.6) | 1205 (61.8) |  |
| Comorbidities |  |  |  | 0.001 |
| None | 1956 (68.0) | 592 (63.9) | 1364 (70.0) |  |
| ≥ 1 | 919 (32.0) | 334 (36.1) | 585 (30.0) |  |
| Lymphovascular invasion |  |  |  | < 0.001 |
| Negative | 2657 (92.4) | 894 (96.5) | 1763 (90.5) |  |
| Positive | 218 (7.6) | 32 (3.5) | 186 (9.5) |  |
| Pathologic tumor size |  |  |  | < 0.001 |
| ≤ 2cm | 1851 (64.4) | 684 (73.9) | 1167 (59.9) |  |
| > 2cm | 1024 (35.6) | 242 (26.1) | 782 (40.1) |  |
| Pathologic nodal status |  |  |  | < 0.001 |
| Negative | 1882 (65.5) | 678 (73.2) | 1204 (61.8) |  |
| Positive | 967 (33.6) | 231 (24.9) | 736 (37.8) |  |
| Unknown | 26 (0.9) | 17 (1.8) | 9 (0.5) |  |
| Histological grade |  |  |  | < 0.001 |
| I-II | 1743 (60.6) | 649 (70.1) | 1094 (56.1) |  |
| III | 665 (23.1) | 54 (5.8) | 611 (31.3) |  |
| Unknown | 467 (16.2) | 223 (24.1) | 244 (12.5) |  |
| Histopathological type |  |  |  | 0.297 |
| Non-IDC | 468 (16.3) | 429 (16.5) | 39 (14.1) |  |
| IDC | 2407 (83.7) | 2169 (83.5) | 238 (85.9) |  |
| ER expression |  |  |  | < 0.001 |
| < 50% | 244 (8.5) | 30 (3.2) | 214 (11.0) |  |
| ≥ 50% | 2631 (91.5) | 896 (96.8) | 1735 (89.0) |  |
| PR status |  |  |  | < 0.001 |
| Negative | 425 (14.8) | 0 (0.0) | 425 (21.8) |  |
| Positive | 2450 (85.2) | 926 (100.0) | 1524 (78.2) |  |
| Ki-67 expression |  |  |  | < 0.001 |
| < 14% | 1310 (45.6) | 926 (100.0) | 384 (19.7) |  |
| ≥ 14% | 1565 (54.4) | 0 (0.0) | 1565 (80.3) |  |
| Surgery of the breast |  |  |  | 0.298 |
| Breast-conserving surgery | 1029 (35.8) | 344 (37.1) | 685 (35.1) |  |
| Mastectomy | 1846 (64.2) | 582 (62.9) | 1264 (64.9) |  |
| Surgery of the axilla |  |  |  | < 0.001 |
| SLNB | 1709 (59.4) | 632 (68.3) | 1077 (55.3) |  |
| ALND | 1140 (39.7) | 276 (29.8) | 864 (44.3) |  |
| Unknown | 26 (0.9) | 18 (1.9) | 8 (0.4) |  |
| Chemotherapy |  |  |  | < 0.001 |
| No | 1262 (43.9) | 655 (70.7) | 607 (31.1) |  |
| Yes | 1613 (56.1) | 271 (29.3) | 1342 (68.9) |  |
| Radiotherapy |  |  |  | < 0.001 |
| No | 1354 (47.1) | 492 (53.1) | 862 (44.2) |  |
| Yes | 1521 (52.9) | 434 (46.9) | 1087 (55.8) |  |
| Endocrine therapy |  |  |  | 0.001 |
| No | 137 (4.8) | 27 (2.9) | 110 (5.6) |  |
| Yes | 2738 (95.2) | 899 (97.1) | 1839 (94.4) |  |
| Ovarian function suppression |  |  |  | < 0.001 |
| No | 2701 (93.9) | 894 (96.5) | 1807 (92.7) |  |
| Yes | 174 (6.1) | 32 (3.5) | 142 (7.3) |  |

Abbreviations: SD, standard deviation; BMI, body mass index; IDC, invasive ductal carcinoma; ER, estrogen receptor; PR, progesterone receptor; SLNB, sentinel lymph node biopsy; ALND, axillary lymph node dissection.

**Supplementary Table S2.** Univariate and multivariate analysis of factors associated with RFS in luminal patients.

| Variables |  | Univariate |  | Multivariate | |
| --- | --- | --- | --- | --- | --- |
|  |  | *P* value |  | HR (95%CI) | *P* value |
| BMI: obese vs non-obese |  | 0.839 |  |  |  |
| Age at diagnosis (year): ≥ 50 vs < 50 |  | 0.586 |  |  |  |
| Menopausal status: post vs pre/peri-menopausal |  | 0.926 |  |  |  |
| Comorbidities: ≥ 1 vs 0 |  | 0.265 |  |  |  |
| LVI: positive vs negative |  | 0.053 |  | 1.17 (0.66-2.07) | 0.597 |
| Pathologic tumor size: > 2cm vs ≤ 2cm |  | <0.001 |  | 1.74 (1.22-2.44) | 0.002 |
| Pathologic nodal status: positive vs negative |  | <0.001 |  | 1.96 (1.35-2.84) | <0.001 |
| Histological grade: III vs I-II |  | <0.001 |  | 1.75 (1.23-2.48) | 0.002 |
| Histopathological type: IDC vs Non-IDC |  | 0.493 |  |  |  |
| ER expression: ≥ 50% vs < 50% |  | 0.024 |  | 1.00 (0.60-1.66) | 0.999 |
| PR expression: positive vs negative |  | <0.001 |  | 0.63 (0.42-0.95) | 0.027 |
| Luminal type: A vs B |  | <0.001 |  | 1.63 (0.95-2.78) | 0.075 |
| Chemotherapy: yes vs no |  | <0.001 |  | 1.34 (0.82-2.17) | 0.244 |
| Radiotherapy: yes vs no |  | 0.050 |  | 1.00 (0.68-1.47) | 0.984 |
| Endocrine therapy: yes vs no |  | <0.001 |  | 0.41 (0.24-0.71) | 0.002 |

**Supplementary Table S3.** Univariate analysis of factors associated with RFS and OS in luminal A patients.

| Variables |  | RFS |  | OS |
| --- | --- | --- | --- | --- |
|  |  | *P* value |  | *P* value |
| BMI: obese vs non-obese |  | 0.021 |  | 0.012 |
| Age at diagnosis (year): ≥ 50 vs < 50 |  | 0.754 |  | 0.276 |
| Menopausal status: post vs pre/peri-menopausal |  | 0.405 |  | 0.062 |
| Comorbidities: ≥ 1 vs 0 |  | 0.520 |  | 0.954 |
| LVI: positive vs negative |  | 0.624 |  | 0.739 |
| Pathologic tumor size: > 2cm vs ≤ 2cm |  | 0.009 |  | 0.445 |
| Pathologic nodal status: |  | 0.018 |  | 0.173 |
| positive vs negative |  |  |  |  |
| unknown vs negative |  |  |  |  |
| Histological grade: |  | 0.209 |  | 0.978 |
| III vs I-II |  |  |  |  |
| unknown vs I-II |  |  |  |  |
| Histopathological type: IDC vs Non-IDC |  | 0.887 |  | 0.752 |
| ER expression: ≥ 50% vs < 50% |  | 0.429 |  | 0.635 |
| Chemotherapy: yes vs no |  | 0.017 |  | 0.306 |
| Radiotherapy: yes vs no |  | 0.239 |  | 0.263 |
| Endocrine therapy: yes vs no |  | 0.581 |  | 0.704 |
| OFS: yes vs no |  | 0.556 |  | 0.688 |

Abbreviations: RFS, relapse-free survival; OS, overall survival; BMI, body mass index; LVI, lymphovascular invasion; IDC, invasive ductal carcinoma; ER, estrogen receptor; OFS, ovarian function suppression.

**Supplementary Table S4.** Univariate and multivariate analysis of factors associated with RFS in luminal B/HER2-negative patients.

| Variables |  | Univariate |  | Multivariate | |
| --- | --- | --- | --- | --- | --- |
|  |  | *P* value |  | HR (95%CI) | *P* value |
| BMI: obese vs non-obese |  | 0.487 |  | 0.78 (0.41-1.49) | 0.454 |
| Age at diagnosis (year): ≥ 50 vs < 50 |  | 0.568 |  |  |  |
| Menopausal status: post vs pre/peri-menopausal |  | 0.504 |  |  |  |
| Comorbidities: ≥ 1 vs 0 |  | 0.577 |  |  |  |
| LVI: positive vs negative |  | 0.112 |  |  |  |
| Pathologic tumor size: > 2cm vs ≤ 2cm |  | < 0.001 |  | 1.81 (1.29-2.56) | 0.001 |
| Pathologic nodal status: |  | < 0.001 |  |  | 0.001 |
| positive vs negative |  |  |  | 2.00 (1.38-2.89) | < 0.001 |
| unknown vs negative |  |  |  | 1.58 (0.21-12.02) | 0.660 |
| Histological grade: |  | < 0.001 |  |  | 0.011 |
| III vs I-II |  |  |  | 1.71 (1.19-2.46) | 0.003 |
| unknown vs I-II |  |  |  | 1.89 (0.46-7.73) | 0.376 |
| Histopathological type: IDC vs Non-IDC |  | 0.077 |  | 0.72 (0.18-2.95) | 0.652 |
| ER expression: ≥ 50% vs < 50% |  | 0.180 |  |  |  |
| Chemotherapy: yes vs no |  | 0.004 |  | 1.30 (0.82-2.05) | 0.266 |
| Radiotherapy: yes vs no |  | 0.211 |  |  |  |
| Endocrine therapy: yes vs no |  | < 0.001 |  | 0.42 (0.25-0.68) | 0.001 |
| OFS: yes vs no |  | 0.492 |  |  |  |

Abbreviations: RFS, relapse-free survival; HR, hazard ratio; CI, confidence interval; BMI, body mass index; LVI, lymphovascular invasion; IDC, invasive ductal carcinoma; ER, estrogen receptor; OFS, ovarian function suppression.

**Supplementary Table S5.** Univariate and multivariate analysis of factors associated with OS in luminal B/HER2-negative patients.

| Variables |  | Univariate |  | Multivariate | |
| --- | --- | --- | --- | --- | --- |
|  |  | *P* value |  | HR (95%CI) | *P* value |
| BMI: obese vs non-obese |  | 0.649 |  | 1.17 (0.50-2.74) | 0.727 |
| Age at diagnosis (year): ≥ 50 vs < 50 |  | 0.057 |  | 2.01 (0.71-5.72) | 0.190 |
| Menopausal status: post vs pre/peri-menopausal |  | 0.090 |  | 1.14 (0.44-2.96) | 0.796 |
| Comorbidities: ≥ 1 vs 0 |  | 0.361 |  |  |  |
| LVI: positive vs negative |  | 0.002 |  | 2.38 (1.12-5.03) | 0.024 |
| Pathologic tumor size: > 2cm vs ≤ 2cm |  | 0.001 |  | 1.72 (1.03-2.88) | 0.040 |
| Pathologic nodal status: |  | < 0.001 |  |  | 0.003 |
| positive vs negative |  |  |  | 2.85 (1.56-5.18) | 0.001 |
| unknown vs negative |  |  |  | 0.00 (0.00-1.48E+179) | 0.966 |
| Histological grade: |  | 0.002 |  |  | 0.133 |
| III vs I-II |  |  |  | 1.73 (0.99-3.03) | 0.055 |
| unknown vs I-II |  |  |  | 2.46 (0.22-28.25) | 0.469 |
| Histopathological type: IDC vs Non-IDC |  | 0.036 |  | 0.60 (0.05-6.80) | 0.680 |
| ER expression: ≥ 50% vs < 50% |  | 0.013 |  | 0.50 (0.27-0.91) | 0.024 |
| Chemotherapy: yes vs no |  | 0.046 |  | 1.27 (0.62-2.61) | 0.521 |
| Radiotherapy: yes vs no |  | 0.341 |  |  |  |
| Endocrine therapy: yes vs no |  | 0.029 |  | 0.45 (0.21-1.00) | 0.049 |
| OFS: yes vs no |  | 0.924 |  |  |  |

Abbreviations: OS, overall survival; HR, hazard ratio; CI, confidence interval; BMI, body mass index; LVI, lymphovascular invasion; IDC, invasive ductal carcinoma; ER, estrogen receptor; OFS, ovarian function suppression.
